# A Novel Method for the Preparation of Retinoic Acid-Loaded Nanoparticles

**DOI:** 10.3390/ijms10052336

**Published:** 2009-05-19

**Authors:** Cesare Errico, Matteo Gazzarri, Federica Chiellini

**Affiliations:** Laboratory of Bioactive Polymeric Materials for Biomedical & Environmental Applications (BIOlab), UdR-INSTM, Department of Chemistry and Industrial Chemistry, University of Pisa, Via Vecchia Livornese 1291, 56122 S. Piero a Grado, Pisa, Italy; E-Mails: cesare@ns.dcci.unipi.it (C.E.); m.gazzarri@ns.dcci.unipi (M.G.)

**Keywords:** polymeric nanoparticles, retinoic acid, colloidal coating

## Abstract

The goal of present work was to investigate the use of bioerodible polymeric nanoparticles as carriers of retinoic acid (RA), which is known to induce differentiation of several cell lines into neurons. A novel method, named “Colloidal-Coating”, has been developed for the preparation of nanoparticles based on a copolymer of maleic anhydride and butyl vinyl ether (VAM41) loaded with RA. Nanoparticles with an average diameter size of 70 nm and good morphology were prepared. The activity of the encapsulated RA was evaluated on SK-N-SH human neuroblastoma cells, which are known to undergo inhibition of proliferation and neuronal differentiation upon treatment with RA. The activity of RA was not affected by the encapsulation and purification processes.

## 1. Introduction

Recently, Tissue Engineering (TE) has become a well recognized technique, and many researchers with different areas of expertise are entering this medical field, thereby increasing its scientific implications. The objective of tissue engineering is to regenerate natural tissues from living cells to replace defective or lost tissues and organs [1].

It is undoubtedly necessary for cell-based tissue regeneration to increase the number of cells that constitute the tissue, as well as reconstruct the structure to support the cells. In addition, growth factors are required to promote cell differentiation and proliferation and thus achieve tissue regeneration. However, few researchers have applied this Drug Delivery System (DDS) to the field of Regenerative Medicine (RM) [2]. Drugs for RM include organic compounds and genes that are effective in promoting the proliferation and differentiation of cells for the induction of tissue and organ regeneration [2].

RA, the main biologically-active derivative of Vitamin A or retinol [3], is a potent regulator of gene transcriptions [4], and play an important role in regulating cell growth, differentiation [3], embryonic development, apoptosis, homeostasis [5] and can reverse malignant cell growth *in vitro* and *in vivo*. All-*trans*-retinoic acid (ATRA) is being increasingly included in antitumour therapeutical schemes for the treatment of various tumoral diseases such as acute promyelocytic leukaemia, Kaposi’s sarcoma, head and neck squamous cell carcinoma, ovarian carcinoma, bladder cancer and neuroblastoma [6] and has shown antiangiogenic effects in several systems [5]. As a result of good clinical responses in treatment of these malignancies, retinoids are being investigated for their therapeutic potential in an increasing range of diseases [3]. However, due to its hydrophobicity [6] (0.21 μM in physiological solution, pH 7.3) [7], and short half-life in blood [8], ATRA parenteral administration is very difficult and to date no commercial parenteral formulation is available. On the other hand the oral formulations in clinical use are characterized by uncertain drug bioavailability due to the variable absorption in the gastrointestinal tract and the hepatic first pass effect, implying metabolism by hepatocytes of ATRA to less active compounds. Furthermore its intestinal absorption is affected by the pH and fatty acid composition of intraluminal bile [9]. Therefore, plasma concentration following oral administration of ATRA is highly variable [6]. In addition to the poor solubility, combined with chemical instability of ATRA complicates its administration.

In spite of its pronounced therapeutical effect, several side effects such as teratogenicety [10], retinoid acute resistance, hypertriglyceridemia, mucocutaneous dryness, headache [11] hepatotoxicity, nausea, vomiting, and abdominal pain [3] are reported and limit the clinical applications of ATRA [8].

Different treatment modalities have been investigated in attempt to overcome the accelerated metabolism of ATRA that leads to a progressive decline in plasma ATRA concentrations during a chronic daily schedule. These modalities include the use of inhibitors of cytochrome p450 enzymes, administration of oral ATRA on an intermittent schedule, and encapsulation of ATRA into microspheres [12], microemulsions [13], nanodisks [14], liposomes [15], micelles [16] and nanoparticles [6,8,9,17,18]. The dimensions of the carrier have a critical role in the achievement of optimized therapeutic regimens. It has been shown that nanocarriers can penetrate through small capillaries, across numerous physiological barriers and can be taken up by cells, thus inducing efficient drug accomulation at the target site [20].

There are a number of techniques available for the preparation of drug loaded nanoparticulate systems such as the emulsion solvent evaporation/extraction method, spray drying, phase separationcoacervation, interfacial deposition, and *in situ* polymerization. Each method has its own advantages and disadvantages. The choice of a particular technique depends on polymer and drug features, site of action and therapy regimes [21].

Although a number of different method exist for the preparation of nanoparticles the choice of a preparation method is an hard task and general procedures that works for all nanoparticles material seem to do not exist. Furthermore it is highly desirable to provide with alternative methods, aimed at producing polymeric particles, that do not entail the utilization of organic solvents that are undesiderable for their potential toxicity. Such a method is expected to receive a particular attention in biotechnological field [21].

In this work an organic solvent-free method has been developed for the preparation of VAM 41 nanoparticles loaded with RA. VAM 41 is an amphiphilic polymeric material developed by hemiesterification of alternating copolymers of maleic anhydride (MAn) and alkyl vinyl ethers (RVE) Quite a large number of examples are reported in the literature in which hemiesters of copolymers of maleic anhydride and alkyl vinyl ethers have been utilized for the formulation of release systems. For instance, the *n*–butyl hemiester of maleic anhydride–methyl vinyl ether alternating copolymer has been used in ophthalmology for the preparation of monolithic systems or as pH sensitive covers of microcapsules [22]. Similarly, alkyl hemiesters of maleic anhydride–oligo(oxyethylene) vinyl ether copolymers were formulated as monolithic inserts for the controlled release of pilocarpine [23], and γ-interferon [22,24].

The developed method is easy to be perform, effective for the preparation of relatively small nanoparticles with a narrow size distribution and is operator friendly, since it does not require handling any toxic organic solvents. The SK-N-SH neuroblastoma cell line was chosen as a model to verify the possibility of differentiating for a neuronal phenotype after exposure to bioactive principles and to examine whether the activity of RA was affected by incorporation in VAM41 nanoparticles by the developed method. The preparation process guarantee for the chemical stability and biological activity of the incorporated drug.

## 2. Results and Discussion

The novel nanoparticle preparation method was conceived by taking into account the characteristics of RA, VAM41 and HSA and the behaviour of their different mixtures in response to pH variations. RA is a hydrophobic compound with one polar, ionisable end group that confers amphiphilic character to the molecule. In non-polar hydrocarbon solvents, RA self associates by forming tail-to-tail dimers that are stabilized by hydrogen bonding between the carboxyl groups of two RA molecules. On the contrary, in water RA self associates in micelle-like structures by hydrophobic interaction among the rings of several molecules [25]. RA at pH > 7.4 and at a 1.5 mM concentration is soluble in water, as evidenced by the clarity of the solution and the lack of any observed light scattering. When the pH is lowered to 7, RA self associates to micellar like structure with a diameter of 120 nm. This phenomenon is macroscopically evidenced by the appearence of turbidity and the size evaluated by granulometry measurements. A further decrease of the solution pH causes the formation of flakes of micelles that leads to RA precipitation. At a pHs below the critical micellar concentration which is about 2 x 10^−6^ mol/L (pH 7) [26], the negative surface charge which stabilize the RA micelles is gradually lost. Particles collide forming aggregates that precipitate.

In presence of HSA (nHSA/nRA 1:10) a stable yellowish milky-like particle suspension with a diameter size of 90 nm forms at pH 7. HSA hampers the precipitation of the drug up to pH 5. However, at pHs below 5 the colloidal suspension looses its stability and tends to agglomerate.

**Figure 1.**
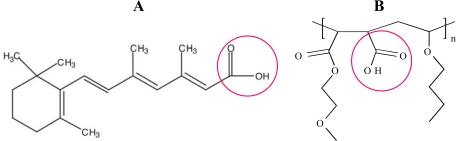
Carboxylic acid moieties confer amphiphilic behaviour to RA (A) and water solubility to VAM41 (B).

This behaviour could be explained by the so-called hydration forces [27]. HSA, once its binding sites for RA are saturated, tends to be adsorbed on the RA colloid’s surface. It is well established that water molecules strongly bind to protein surfaces. An overlap of the solvent layers near to the two mutually approaching colloid surfaces creates repulsive forces [28]. At pH 5, a pH lower than the isoelectric point (pI) of HSA, protein molecules have a compact coil structure and an electrostatic attraction force favours the protein (positively charged) surface union [27] which leads to the loss of colloidal stability of the system. VAM41, due to the presence of the ionisable carboxylate group is soluble in water at pHs above 8 and it is insoluble at pHs below 7 (Figure 1). HSA, as previously reported, is able to complex RA and stabilize suspension colloidal particles, is soluble at all mentioned pHs, and due to the presence of many charged residues on the molecule, it has buffering properties. At physiological pH (7.4) it displays a net negative charge [29].

**Figure 2.**
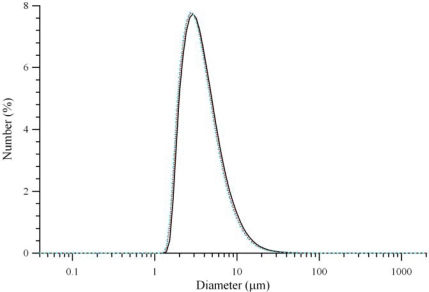
Particle size distribution obtained from a water solution containing VAM41 and HSA.

It was observed that the addition of a drop of 1N chloride acid to a water solution of VAM41 and HSA at pH 8 led, as expected, to the formation of polymer flakes. Unexpectedly, after few seconds under energetic stirring, these flakes completely re-dissolved. HSA, thanks to its buffering properties, was able to deprotonate the carboxylic moieties of VAM41. The further addition of drops of acid led to a slow decrease of the pH of the solution, to a reduction of the ability of HSA to accept protons and a milk-like colloidal suspension of VAM41 formed at about pH 6. At pH 5.5 a colloidal suspension of VAM41 with average diameter size of the particles of 2.74 μm (polydispersity: 0.3) and a rather wide distribution of the diameters (Figure 2) was observed that became 4.64 μm at pH 2.5.

On the basis of this observation and keeping in mind the characteristics of the material employed, a water solution of VAM41, RA and HSA was prepared by adding NaOH to a water suspension of the materials. The solution pH was adjusted to 8 and subsequently drops of HCl 1 N and 0.1 N, as in a typical titration experiment, were added under energetic stirring in order to prepare nanoparticles. As already experienced, the addition of drops of acid to the solution led to the formation of flakes that rapidly re-dissolved. As hypothesized, at roughly pH 7.4 micelles of RA formed, while the polymer kept re-dissolving in the same fashion. The subsequent additions of acid caused the polymer to absorb and coat uniformly the surface of the formed colloidal suspension. The energetic stirring was essential for a uniform distribution of the polymer onto RA micelles. HCl 1 N was used in the first part of the experiment when the dissolution of the forming flakes was quite fast. Once the buffering properties of HSA were reduced, HCl 0.1 N was used to terminate the covering process. RA acted as nucleating agent during the preparation of nanoparticle suspensions.

The colloidal suspension was stable up to pH 5.3. Below this pH particles joined together forming a dense aggregate that could be uniformly redispersed just by adding drops of dilute NaOH. The formation of aggregates could be ascribed to the above mentioned loss of proteins stabilizing effect at pHs close or below to their pI. Particle formation and development was followed by pHmeter and light scattering (Table 1).

**Figure 3.**
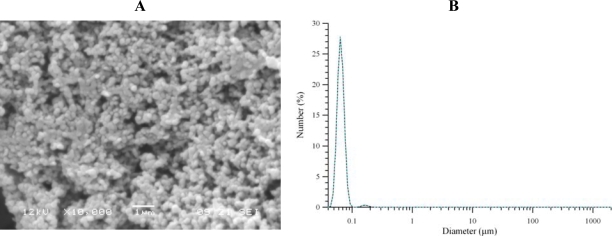
Morphology (A) and particle size distribution (B) of RA loaded nanoparticles prepared by Colloidal Coating (pH 5.3).

Nanoparticles with good morphology and an average diameter size of about 65 nm were prepared using this technique (Figure 3).

Moreover a drug loading content of 6.7% with a loading efficiency of 97% was obtained when the experiment was terminated at pH 5.3. Drug loading efficiency is very high and depends on the extent of acidification of the solution, since the lower is the pH reached, the higher is the amount of polymer that covers and forms nanoparticles. This formulation method is basically suitable for any material with water solubility influenced by the pH.

During the preparation of nanoparticles by Colloidal Coating, RA is subjected to pH variations, freezing and drying process. In order to examine whether the activity of RA was affected by incorporation in VAM41 nanoparticles, it was tested on SK-N-SH human neuroblastoma cell line that are known to undergo inhibition of proliferation and neuronal differentiation upon treatment with RA. SK-N-SH neuroblastoma cell line was chosen as a model to verify the possibility to differentiate towards a neuronal phenotype after exposure to bioactive principles.

**Figure 4.**
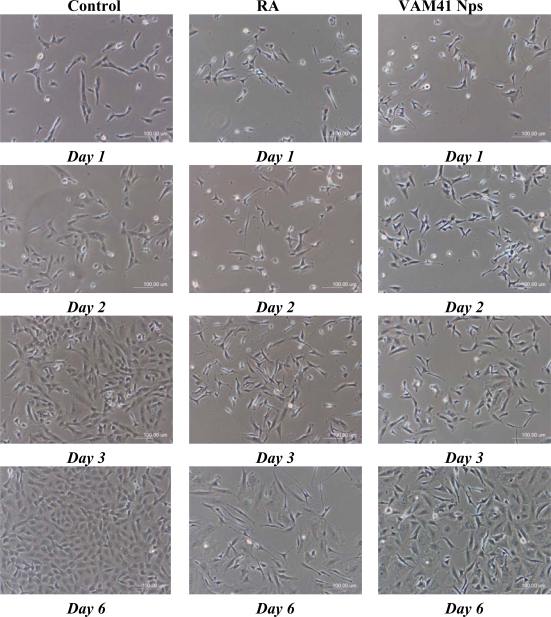
Bright Field Microscopy evalution of SK-N-SH morphology in samples untreated (negative control), treated with free RA (RA positive control) and with nanoparticles loaded with RA (VAM41 Nps).

Numerous studies have shown that RA induces differentiation, growth arrest, and apoptosis in neuroblastoma cell lines by inhibition of telomerase activity, and in increased endogenous ceramide levels [30]. Results of the qualitative investigations of SK-N-SH cells treated with RA loaded nanoparticles appear to indicate a growth inhibition in respect to the untreated cells (negative control) (Figure 4), thus suggesting that the antiproliferative and differentiating activity of RA was not impaired by the nanoparticles incorporation and freeze-drying process. Observation of cells treated with free RA indicates a higher growth inhibition activity in respect to the RA loaded into nanoparticles. It is reasonable to speculate that this result is related to a controlled release of the bioactive agent RA from nanoparticles.

## 3. Experimental Section

### 3.1. Materials

The bioerodible polymer VAM41 [2-methoxyethanol hemiester of poly(maleic anhydride-alt-butyl vinyl ether)] was kindly provided by Polymer Laboratories Ltd (UK). Retinoic acid (RA) and human serum albumin (HSA, 96 – 99% powder) were purchased from Sigma (St. Louis, MO, USA). A 3% mannitol solution was prepared by dissolving 3.0 g of mannitol (Baker) in 100 mL distilled water (Phillipsburg, NJ, USA). Hydrochloric Acid 37 – 38% the commercial product (J.T. Baker, Philipsburg, NJ, USA) was used as received and diluted with deionised water to prepare 1 N and 0.1 N acid solutions. SK-N-SH human neuroblastoma cell line (HTB-11) was purchased from American Type Culture Collection (ATCC, Rockville, MD) and propagated as indicated by the supplier. Minimum Essential Medium (MEM), 0.01 M phosphate buffer saline (pH 7.4) free of Ca^2+^ and Mg^2+^ (PBS), fetal calf serum, glutamine and antibiotics (penicillin/streptomycin) were purchase from GIBCO Brl (Rockville, MD).

### 3.2. Preparation of Unloaded Nanoparticles

50 mg of VAM41 were dissolved in 5 mL of water by adding a drop 10 N NaOH and then 200 μL of 25 % HSA solution (corresponding to 50 mg HSA) were added. The solution was left under stirring conditions for 1 h and the pH was adjusted to 8. At room temperature and under rapid magnetic stirring (7,500 rpm) 1 N HCl was dropped into the solution until the formation of a suspension (~ pH 5). Each drop was added after the disappearing of the flakes formed by the addition of the previous drop.

### 3.3. Preparation of RA Loaded Nanoparticles

50 mg of VAM41 were dissolved in 5 mL of water by adding a drop 10 N NaOH and then 200 μL of 25 % HSA solution (corresponding to 50 mg HSA) were added and the solution was kept under stirring condition for 1 h. Subsequently 225 μL of a solution of retinoic acid in DMSO (10 mg/mL) were added and the pH was adjusted to 8. At room temperature and under rapid magnetic stirring 1 N HCl was dropped into the solution until the formation of a yellowish suspension (~ pH 7.4). Each drop was added after the disappearing of the flakes formed by the addition the previous drop. In the same fashion drops of 1 N and 0.1 N HCl were added up to pH 5.3.

### 3.4. Nanoparticles Purification

Nanoparticle suspensions were purified by centrifugation in an ALCPK121R centrifuge at 8,000 g for 30 minutes at room temperature in order to remove the excess of polymer, drug and protein. The samples were either placed in 50 mL falcon or in Eppendorf tubes and centrifuged by using AM-10 or AM-21 rotors, respectively. Harvested nanoparticles were freeze-dried in a 5Pascal Lio 5P lyophilizator.

### 3.5. Cryoprotection

Nanoparticle suspensions (6.0 mL) were centrifuged at 8,000 g for 30 minutes. The resulting pellets were dispersed in 3.0 mL of aqueous solutions containing 3 % mannitol. The resulting suspensions were frozen at −20 °C, freeze-dried in a 5Pascal Lio 5P lyophilizator. The recovered lyophilized powders were re-dispersed in 3.0 mL of deionised water.

### 3.6. Nanoparticle Characterization

Size Analysis: dimensional analyses were carried out by using a Coulter LS230 Laser Diffraction Particle Size Analyzer, equipped with small volume module plus. Nanoparticle suspensions were added into the cell until 30 – 50% obscuration of PIDS detector was reached. Deionised water was used as background and diameter distribution was processed using the Fraunhofer optical model. Three runs were performed on each sample.

Morphological Analysis: nanoparticles morphology was investigated by means of scanning electron microscopy (SEM), by using a JEOL LSM5600LV scanning electron microscope. Gold sputtering was performed before SEM analysis.

### 3.7. Evaluation of Retinoic Acid Incorporation

Samples of purified and lyophilised VAM41 nanoparticles were weighted properly and dissolved into 5 mL of water by adding 1 N NaOH. The final pH was adjusted to 8. Drug concentration was determined spectrophotometrically [31] at 347 nm by using a standard curve obtained by plotting the average blank-corrected absorbance of each standard vs. its RA concentration (0.04 – 10 mg/L; y = 0.1823x + 0.0.0051; R^2^ = 0.9994).

### 3.8. In Vitro Evaluation of Encapsulated Retinoic Acid Activity

SK-N-SH human neuroblastoma cells were seeded in 6 multi-well plates (1.5 × 10^4^ cells/well) and maintained in Minimum Essential Medium (MEM) containing 10% fetal calf serum, 4 mM glutamine and 100 U/mL:100 μg/mL penicillin:streptomycin at 37 °C in 5% CO_2_ for 24 h. Cells were treated either with 40 μM free-RA or 40 μM encapsulated RA for 6 days. Encapsulated or free RA was added to the cells in fresh growth media at every 48 h and morphological differentiation assessed by phase contrast microscopy, by using a Nikon TE-2000 inverted microscope.

## 4. Conclusions

An organic solvent-free method, called “*Colloidal-Coating*”, has been developed for the preparation of VAM 41 nanoparticles loaded with RA. This method presents numerous advantages when compared with the other nanoparticles preparation methods. It does not entail the utilization of toxic organic solvents with obvious implications from a biocompatibility point of view, it is easy to perform, allows for the formulation of small nanoparticles with narrow size distribution, it is based on a reversible process and wastes of material in case of accidental errors during the preparation are avoided. It is possible to prepare multi-layer nanocapsules by adding at different stages of the preparation functionalized polymers and might be extended for the preparation of ferrofluids or in general to encapsulate any substance that can act as nucleating agent. The activity of RA encapsulated by colloidal-coating method was evaluated. The data obtained suggested that the antiproliferative and differentiating activity of RA was not impaired by incorporation and purification processes. SK-N-SH neuroblastoma cell line was chosen as a model to verify the possibility to differentiate towards a neuronal phenotype after exposure to bioactive principles.

## Figures and Tables

**Table 1.** Correlation between pH, size and macroscopic appearance of nanoparticles suspensions prepared by Colloidal-Coating.

**pH**	**Diameter (nm) number % ± SD**	**Polydispersity Index**	**Macroscopic observations**
8	-	-	yellowish solution
7.5	59 ± 11	0.1	slight yellowish milky like suspension
6.2	n.d.	n.d.	dense yellowish milky like suspension
5.2	87 ± 44	0.2	presence of agglomerates in solution
5.7	78 ± 17	0.1	yellowish milky like suspension
5.3	69 ± 14	0.1	dense yellowish milky like suspension

## References

[1] Tabata Y Recent progress in tissue engineering Drug DiscovToday2001 6 483 487 10.1016/s1359-6446(01)01753-6 11344034

[2] Tabata Y Significance of release technology in tissue engineering Drug DiscovToday2005 10 1639 1646 10.1016/S1359-6446(05)03639-1 16376824

[3] Armstrong JL, Redfern CPF, Veal GJ (2005). 13-cis Retinoic acid and isomerisation in paediatric oncology—is changing shape the key to success?. Biochem. Pharmacol.

[4] Noy N (2000). Retinoid-binding proteins : mediators of retinoid action. Biochem. J.

[5] Maiti TK, Ghosh KS, Debnath J, Dasgupta S (2006). Binding of all-trans retinoic acid to human serum albumin: Fluorescence, FT-IR and circular dichroism studies. Int. J. Biol. Macromol.

[6] Zuccari G, Carosio R, Fini A, Montaldo PG, Orienti I (2005). Modified polyvinylalcohol for encapsulation of all-trans-retinoic acid in polymeric micelles. J. Control. Rel.

[7] Szuts EZ, Harosi FI (1991). Solubility of retinoids in water. Arch. Biochem. Biophys.

[8] Jeong YI, Kang MK, Sun HS, Kang SS, Kim HW, Moon KS (2004). All-trans-retinoic acid release from core-shell type nanoparticles of poly(ε-caprolactone)/poly (ethylene glycol) diblock copolymer. Int. J. Pharm.

[9] Ozpolat B, Lopez-Berestein G, Adamson P, Fu CHJ, Williams AH (2003). Pharmacokinetics of intravenously administered liposomal all-trans-retinoic acid (atra) and orally administered atra in healthy volunteers. J. Pharm. Pharmaceut. Sci.

[10] Hendrickx AG, Hummler H (1991). Teratogenicity of all-trans retinoic acid during early embryonic development in the cynomolgus monkey (Macaca fascicularis). Teratology.

[11] Jeong YI, Song JG, Kang SS, Ryu HH, Lee YH, Choi C (2003). Preparation of poly(DL-lactide-co-glycolide) microspheres encapsulating all-trans retinoic acid. Int. J. Pharm.

[12] Park K, Yang JH, Choi Y, Lee C, Kim SY, Byun Y (2005). Chemoprevention of 4-NQO-induced oral carcinogenesis by co-administration of all-trans retinoic acid loaded microspheres and celecoxib. J. Control. Rel.

[13] Hwang SR, Lim SJ, Park JS, Kim CK (2004). Phospholipid-based microemulsion formulation of all-trans-retinoic acid for parenteral administration. Int. J. Pharm.

[14] Redmond KA, Nguyen TS, Ryan RO (2007). All-trans-retinoic acid nanodisks. Int. J. Pharm.

[15] Kawakami S, Opanasopit P, Yokoyama M, Chansri N, Yamamoto T, Okano T, Yamashita F, Hashida M (2005). Biodistribution characteristics of all-trans retinoic acid incorporated in liposomes and polymeric micelles following intravenous administration. J. Pharm. Sci.

[16] Chansri N, Kawakami S, Yokoyama M, Yamamoto T, Charoensit P, Hashida M (2007). Anti-tumor effect of all- trans retinoic acid loaded polymeric micelles in solid tumor bearing mice. Pharm. Res.

[17] Newman KD, McBurney MW (2004). Poly(d,l lactic-*co*-glycolic acid) microspheres as biodegradable microcarriers for pluripotent stem cells. Biomaterials.

[18] Hwang SR, Lim SJ, Park JS, Kim CK (2004). Phospholipid-based microemulsion formulation of all-trans-retinoic acid for parenteral administration. Int. J. Pharm.

[19] Chiellini F, Piras AM, Errico C, Chiellini E (2008). Micro/nanostructured polymeric systems for biomedical and pharmaceutical applications. Nanomedicine.

[20] Sinha VR, Bansal K, Kaushik R, Kumria R, Trehan A (2004). Poly-epsilon-caprolactone microspheres and nanospheres:an overview. Int. J. Pharm.

[21] Chenite A, Selmani A Method for preparation of polymer microparticles free of organic solvent traces.

[22] Chiellini E, Leonardi G, Giannasi D, Solaro R (1992). Partial esters of alternating copolymers of maleic anhydride and alkyl vinyl ethers for pharmaceutical applications. J. Bioact. Compat. Polym.

[23] Chiellini E, Saettone MF (1988). Polymer drug delivery systems in ophthalmic applications. J. Bioact. Compat. Polym.

[24] Chiellini E, Solaro R, Leonardi G, Giannasi D, Lisciani R, Mazzanti G (1992). New polymeric hydrogel formulations for the controlled release of α-interferon. J. Control. Rel.

[25] Noy N (1992). The ionization behaviour of retinoic acid in aqueous environments and bound to serum albumin. Biochim. Biophis. Acta.

[26] Thünemann AF, Beyermann J, Kukula H (2000). Poly (ethylene oxide)-b-poly(L-lysine) complexes with retinoic acid. Macromolecules.

[27] Molina-Bolìvar JA, Ortega-Vinuesa JL (1999). How proteins stabilize colloidal particles by means of hydration forces. Langmuir.

[28] Molina-Bolìvar JA, Galisteo-Gonzàlez F, Hidalgo-Alvarez R (1997). Colloidal stability of protein-polymer systems: A possible explanation by hydration forces. Phys. Rev. E.

[29] Nicholson JP, Wolmarans MR, Park GR (2000). The role of albumin in critical illness British. J Anaesth.

[30] Kraveka JM, Li L, Bielawski J, Obeid LM, Ogretmen B (2003). Involvement of Endogenous ceramide in the inhibition of telomerase activity and induction of morphologic differentiation in response to all-trans-retinoic acid in human neuroblastoma cells. Arch. Biochem. Biophys.

[31] Karnaukhova E (2007). Interactions of human serum albumin with retinoic acid, retinal an retinyl acetate. Biochem. Pharmacol.

